# The Dual Role of the GABA_A_ Receptor in Peripheral Inflammation and Neuroinflammation: A Study in Hyperammonemic Rats

**DOI:** 10.3390/ijms22136772

**Published:** 2021-06-24

**Authors:** Michele Malaguarnera, Tiziano Balzano, Mari Carmen Castro, Marta Llansola, Vicente Felipo

**Affiliations:** 1 Laboratory of Neurobiology, Centro de Investigación Príncipe Felipe, 46012 Valencia, Spain; michele.malaguarnera@gmail.com (M.M.); tbalzano.hmcinac@hmhospitales.com (T.B.); mcastro@cipf.es (M.C.C.); vfelipo@cipf.es (V.F.); 2 Department of Psychobiology, Facultad de Psicología, Universitat de Valencia, 46010 Valencia, Spain; 3 HM CINAC (Centro Integral de Neurociencias Abarca Campal), Hospital Universitario HM Puerta del Sur, HM Hospitales, 28938 Madrid, Spain

**Keywords:** bicuculline, cerebellum, astrocyte activation, microglia phenotype, cytokines, GABA and glutamate transporters, hepatic encephalopathy

## Abstract

Cognitive and motor impairment in minimal hepatic encephalopathy (MHE) are mediated by neuroinflammation, which is induced by hyperammonemia and peripheral inflammation. GABAergic neurotransmission in the cerebellum is altered in rats with chronic hyperammonemia. The mechanisms by which hyperammonemia induces neuroinflammation remain unknown. We hypothesized that GABA_A_ receptors can modulate cerebellar neuroinflammation. The GABA_A_ antagonist bicuculline was administrated daily (i.p.) for four weeks in control and hyperammonemic rats. Its effects on peripheral inflammation and on neuroinflammation as well as glutamate and GABA neurotransmission in the cerebellum were assessed. In hyperammonemic rats, bicuculline decreases IL-6 and TNFα and increases IL-10 in the plasma, reduces astrocyte activation, induces the microglia M2 phenotype, and reduces IL-1β and TNFα in the cerebellum. However, in control rats, bicuculline increases IL-6 and decreases IL-10 plasma levels and induces microglial activation. Bicuculline restores the membrane expression of some glutamate and GABA transporters restoring the extracellular levels of GABA in hyperammonemic rats. Blocking GABA_A_ receptors improves peripheral inflammation and cerebellar neuroinflammation, restoring neurotransmission in hyperammonemic rats, whereas it induces inflammation and neuroinflammation in controls. This suggests a complex interaction between GABAergic and immune systems. The modulation of GABA_A_ receptors could be a suitable target for improving neuroinflammation in MHE.

## 1. Introduction

Patients with liver cirrhosis may develop covert or minimal hepatic encephalopathy (MHE) characterized by mild cognitive impairment, attention deficits, and psychomotor slowing, which impair quality of life, reduce life span, and increase the risks of accidents, falls, and hospitalization. MHE affects several million people and is a serious health, social, and economic problem [1].

Hyperammonemia and peripheral inflammation play synergistic roles in inducing cognitive and motor alterations in MHE [2,3,4]. 

Chronic hyperammonemia per se is enough to induce neuroinflammation with the activation of microglia increasing inflammatory markers in the brain associated with the impairment of cognitive function. Reducing neuroinflammation with ibuprofen restored the learning of a discrimination task in the Y maze in hyperammonemic rats. In addition, chronic hyperammonemia also induces peripheral inflammation, increasing TNFα and decreasing IL-10 levels in the plasma [5]. Reducing peripheral inflammation with anti- TNFα reduces cerebellar neuroinflammation by mechanisms involving the inhibition of the activation of TNFR1-NFkB and associated signaling pathways [6].

Hyperammonemic rats show increased GABAergic tone in the cerebellum. Chronic treatment with bicuculline, a GABA_A_ receptor antagonist, restores GABAergic tone, the function of the glutamate-nitric oxide-cGMP pathway in the cerebellum, and learning of the discrimination task in the Y maze modulated by this pathway [7]. The same effects are induced by treatment with pregnenolone sulphate, a negative allosteric modulator of the GABA_A_ receptor, which also improves the motor incoordination caused by increased extracellular GABA in the cerebellum [8]. In addition, an antagonist of allopregnanolone, GR3027, which also antagonizes the GABA_A_ receptor, restores motor incoordination in rats with portacaval anastomosis and in hyperammonemic rats.

Decreasing neuroinflammation reverses the increase of GABAergic tone in the cerebellum and restores impaired motor coordination in rats with hyperammonemia or with porta-cava anastomosis, suggesting that neuroinflammation alters GABA neurotransmission in the cerebellum of these rats [9,10,11]. 

The presence of GABA_A_ receptors in lymphocytes led to the study of its role in the immune system. Recently, the anti-inflammatory effects of the activation of these receptors was reported [12,13,14]. GABA acts as an anti-inflammatory agent in rheumatoid arthritis, downregulating the mechanisms that lead to the production of pro-inflammatory agents such as IL-1β [15]. The activation of GABA_A_ receptors also ameliorates inflammatory damage in models of multiple sclerosis [13]. GABA acts as an anti-inflammatory agent, decreasing LPS-induced activation of astrocytes and microglia and inhibiting proinflammatory pathways and the increase in proinflammatory cytokines, partially through the activation of GABA_A_ receptors in microglia and astrocytes [16]. The protective role of GABA_A_ activation in LPS-induced inflammation, neuroinflammation, and neurologic alterations was also widely studied [17,18,19,20].

In contrast, some reports suggested that GABA can induce pro-inflammatory cytokine production in pathological conditions. Carmans et al. [17] showed that exogenous systemic administration of GABA increases IL-6 and TNFα mRNA in the central nervous system. Sallam et al. [18] reported that the intracerebral administration of bicuculline inhibited the increase in serum IL-6 and TNFα induced by LPS in rats. Increased GABA levels and the subsequent activation of GABA_A_ receptors induce the activation of astrocytes [21]. 

Our previous work showed the anti-inflammatory effects of systemic administration of bicuculline in the hippocampus of hyperammonemic rats associated with the improvement of spatial learning and anxiety [22].

We proposed that there is an interplay between neuroinflammation and GABAergic and glutamatergic neurotransmission in the induction of cognitive and motor alterations in rats with MHE [23].

It is necessary to note that the effect of systemic modulation of GABA_A_ could be the result of the modulation of GABA_A_ receptors in both peripheral immune cells and cells in the brain.

The aim of this work was to analyze the effects of blocking GABA_A_ receptors on peripheral inflammatory and neuroinflammatory markers in the cerebellum of control and hyperammonemic rats as well as its effects on GABA and glutamate neurotransmission. 

We used bicuculline as GABA_A_ receptor antagonist, as our previous reports showed effects of this compound on cerebellar-dependent learning and signal transduction pathways associated with glutamate NMDA receptors in hyperammonemic rats and we know the effective dose.

## 2. Results

**Figure 1:**
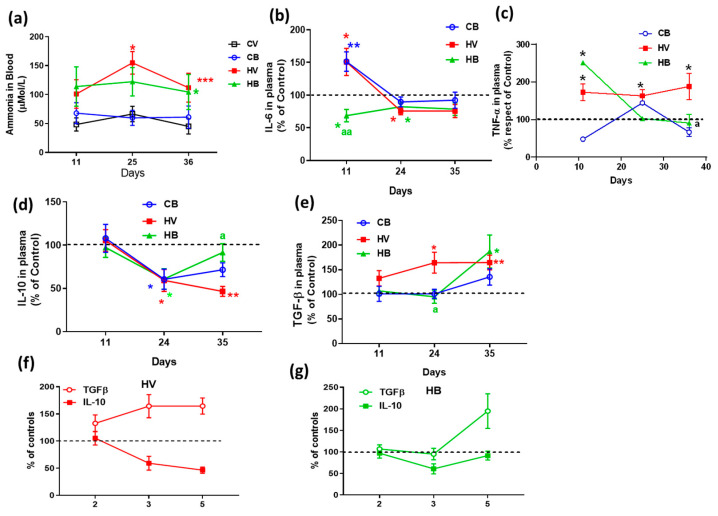
Effect of bicuculline on ammonia in blood and peripheral markers of inflammation. (**a**) Ammonia concentration in the blood. (**b**) Time course of IL-6 levels in the plasma. (**c**) TNFα levels in the plasma after five weeks of hyperammonemia, expressed as percentage of controls. (**d**) Time course of plasmatic IL-10 levels. (**e**) Time course of TGF-β levels in plasma. Cytokine levels are expressed as a percentage of controls. (**f**,**g**) The time course of change in IL-10 and TGF-β plasmatic levels in hyperammonemic rats without (**f**) and with bicuculline treatment (**g**). Values are the mean ± SEM of 18–22 rats/group for ammonia, 5–7 rats for IL6, 6–9 rats for TNFα, 7–10 rats for IL-10, and 6–10 rats for TGF-β. Values significantly different from the control rats are indicated by asterisks. * *p* < 0.05; ** *p* < 0.01; *** *p* < 0.001. Values significantly different from hyperammonemic rats are indicated by “a”. a, *p* < 0.05, and aa, *p* < 0.01. CV, control vehicle; CB, control treated with bicuculline; HA, hyperammonemic rats with vehicle; HB, hyperammonemic rats treated with bicuculline.

We checked whether the systemic administration of bicuculline affected hyperammonemia. Bicuculline administration did not modify the blood ammonia level in hyperammonemic rats, which ranged between 100 and 150 µM, whereas, in control rats, ammonia levels in blood were around 50 µM. Two-way ANOVA with repeated measures was performed (F (3, 58) = 6.234, *p* < 0.001). No significant time effect was found; the *p*-value between control and hyperammonemic curves was *p* < 0.001 and was *p* < 0.05 between hyperammonemia with bicuculline and controls, with no significant effect of bicuculline on the ammonia levels in hyperammonemic rats (Figure 1a).

As previous studies of our group reported peripheral inflammation in hyperammonemic rats with a transitory increase in the levels of IL-6 early after start of the hyperammonemic diet and sustained increase in plasma TNFα, we analyzed the effect of bicuculline systemic administration on these parameters. 

Hyperammonemic rats showed increased levels of IL-6 (151 ± 15% of control, *p* < 0.05) in the plasma at 11 days of hyperammonemia but not thereafter. Bicuculline prevented the early increase in IL-6 in the plasma of hyperammonemic rats (69 ± 9% of controls, *p* < 0.01), whereas, in control rats, bicuculline significantly increased the content of IL-6 (151 ± 21% of control, *p* < 0.01) (Figure 1b). Two-way ANOVA analysis showed a significant time effect, F (1.705, 30.68) = 14.09, group effect, F (2, 29) = 6.155 and interaction, F (4, 36) = 5.609. 

The plasmatic levels of TNFα were significantly increased in hyperammonemic rats throughout the experiment. Bicuculline administration reduced TNFα levels after three or five weeks of the hyperammonemic diet but not after only three days of bicuculline treatment (11 days of hyperammonemia), suggesting an effect of the chronic administration of bicuculline rather than an acute effect. At five weeks of hyperammonemia, TNFα levels were increased by 217 ± 44% compared to controls (*p* < 0.05), and bicuculline chronic administration significantly decreased TNFα levels in hyperammonemic rats, returning to control levels (91 ± 23% of controls, *p* < 0.05 compared with hyperammonemia without bicuculline). F (3, 15) = 5.559 and *p* < 0.01 were the values for two-way ANOVA (Figure 1c).

To better assess the effects of bicuculline on inflammatory parameters in the plasma, we also analyzed the levels of some anti-inflammatory cytokines. IL-10 in the plasma of hyperammonemic rats was significantly decreased after three or five weeks of hyperammonemia (59 ± 13 and 47 ± 6% of control, respectively). Two-way ANOVA with repeated measures showed a significant time effect with F (1.4, 61.87) = 11.34, *p* < 0.001. Bicuculline increased IL-10 after five weeks of hyperammonemia (92 ± 10% of controls, *p* < 0.05) but not after three weeks (61 ± 12% of controls). In control rats, bicuculline significantly decreased IL-10 in plasma to 61 ± 12 and 72 ± 8% of controls after two or four weeks of chronic administration, respectively, *p* < 0.05 for both (Figure 1d).

The plasmatic levels of TGF-β were also significantly increased after three or five weeks of hyperammonemia (164 ± 21 and 165 ± 15% of controls, respectively). Bicuculline decreased the levels of TGF-β in hyperammonemic rats after three (95 ± 13% of controls, *p* < 0.05) but not five weeks of hyperammonemia (187 ± 33% of controls) (Figure 1e). Statistical values in two-way ANOVA with repeated measures were F (1.669, 22.53) = 7.131, *p* < 0.01 for time effect and F (2, 31) = 4.632, *p* < 0.05 for group effect.

The activation of regulatory T lymphocytes increased the levels of the anti-inflammatory cytokines IL-10 and TGF-β. However, in hyperammonemic rats, IL-10 decreased whereas TGF-β increased, indicating that the changes in levels of these cytokines in hyperammonemic rats were not due to the activation of regulatory T-cells but to the modulation of other cell types or mechanisms modulating the plasmatic levels of these cytokines. However, in hyperammonemic rats treated with bicuculline, the levels of both cytokines increased after five weeks of hyperammonemia, suggesting that chronic bicuculline can activate the regulatory T-cells that increase IL-10 and TGF-β levels in the plasma of hyperammonemic rats, whereas, in control rats, plasmatic levels of IL-10 were decreased by bicuculline chronic administration (Figure 1f,g).

**Figure 2:**
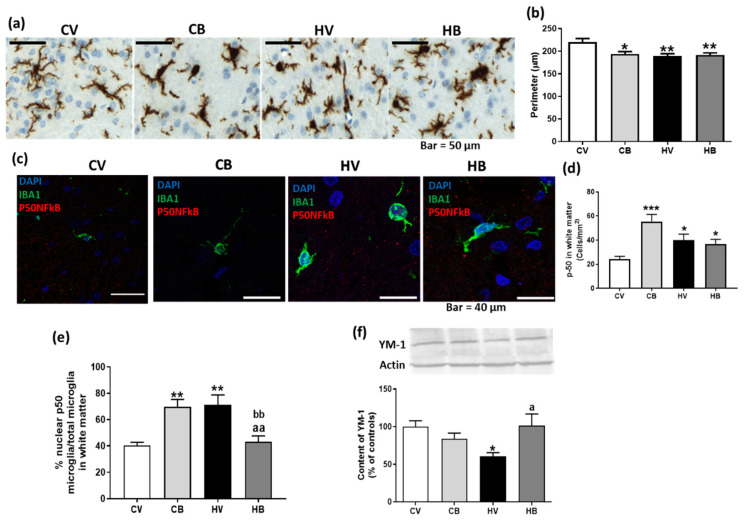
Effect of bicuculline on microglia phenotype in white matter of the cerebellum. Microglia was marked with anti-Iba1. Representative images of microglial morphology (**a**) are shown. The perimeter of microglia was quantified as a measure of microglial activation (**b**). The expression of NFkB p50 subunit in microglia is shown in (**c**) and its quantification in (**d**). The nuclear localization of the p50 subunit of NFkB was quantified from immunofluorescence (**e**). Microglia M2 marker YM-1 was quantified by Western blot and expressed as a percentage of controls (**f**). Data are the mean ± SEM of 4–5 rats for microglia analysis, 3–4 rats for NFkB p50 analysis, and 8–10 rats per group for YM-1 quantification. Values significantly different from control rats are indicated by asterisks. * *p* < 0.05, ** *p* < 0.01, *** *p* < 0.001. Values significantly different from HV rats are indicated by a. a *p* < 0.05, aa, *p* < 0.01. And significantly different from CB are indicated by b. bb *p* < 0.01. CV, control vehicle; CB, control treated with bicuculline; HV, hyperammonemic rats with vehicle; HB, hyperammonemic rats treated with bicuculline.

**Figure 3:**
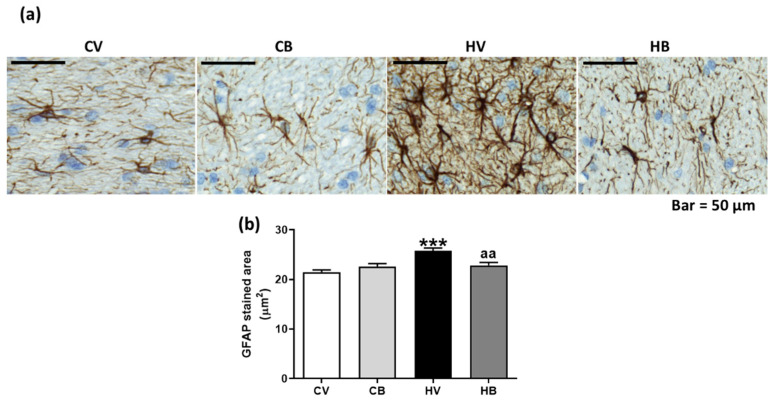
Effect of bicuculline on astrocyte activation in white matter of the cerebellum. Astrocytes were stained with anti-GFAP. Representative images are shown in (**a**). The GFAP-stained area was quantified (**b**). Data are the mean ± SEM of four rats per group. Values significantly different from control rats are indicated by asterisks. *** *p* < 0.001. Values significantly different from hyperammonemic rats are indicated by “aa”, *p* < 0.01. CV, control vehicle; CB, control treated with bicuculline; HV, hyperammonemic rats with vehicle; HB, hyperammonemic rats treated with bicuculline.

As peripheral inflammation induces neuroinflammation in the cerebellum of hyperammonemic rats, we evaluated the effect of bicuculline on cerebellar neuroinflammation. The activation of microglia was assessed by immunohistochemistry, with the microglial marker Iba1 used to analyze the morphology by measuring the perimeter of stained cells. In hyperammonemic rats, microglia displayed a more amoeboid morphology with shorter prolongations. Bicuculline-treated rats, both control and hyperammonemic, also showed microglia with an amoeboid form (Figure 2a). These results were confirmed by perimeter measurements. A decrease in perimeter indicated a change in microglia morphology after activation. The microglial perimeter was significantly shorter in hyperammonemic (189 ± 5 µm, *p* < 0.01) than in control rats (221 ± 8 µm). Bicuculline did not reverse the activation of microglia in hyperammonemic rats (192 ± 5 µm, *p* < 0.01) and significantly decreased the perimeter of microglia in control rats (194 ± 6 µm, *p* < 0.05), indicating their activation. One-way ANOVA parameters in Figure 3B were: F (3, 5) = 4.875, *p* < 0.01 (Figure 2b).

The activation of microglia is associated with the induction of the NFkB transcription factor in hyperammonemic rats, demonstrated by the increased nuclear localization of P50 NFkB in cerebellar microglia [6]. The expression of NFkB was significantly increased in microglia of hyperammonemia rats and also in rats treated with bicuculline, both control and hyperammonemic (F (3, 8) = 8.542, *p* < 0.001). However, the nuclear percentage of P50 NFkB was significantly increased in hyperammonemic rats (62 ± 7%, *p* < 0.01) compared with controls (25 ± 4% in nucleus), but the treatment with bicuculline significantly decreased the percentage of P50 NFkB in the nucleus in hyperammonemic rats (35 ± 7%, *p* < 0.05), while, in control rats, it significantly increased nuclear P50 NFkB (62 ± 7%, *p* < 0.01). One-way ANOVA parameters were: F (3, 7) = 7.515, *p* < 0.001. These results suggest that, although the microglia morphology was not changed by bicuculline, it decreased NFkB activation in hyperammonemic rats (Figure 2c–e). We assessed whether this was due to a change in the microglia phenotype to anti-inflammatory or regulatory M2. In order to assess this possibility, we quantified the content of the M2 marker YM-1. The content of YM-1 was significantly decreased (60 ± 5% of controls, *p* < 0.05) in hyperammonemic rats, whereas it did not decrease in hyperammonemic rats treated with bicuculline (101 ± 16% of controls, *p* < 0.05 compared with hyperammonemia without bicuculline): F (3, 36) = 4.057, *p* < 0.05. Therefore, bicuculline should induce an M2 phenotype in the cerebellar microglia of hyperammonemic rats (Figure 2f). 

We also analyzed the astrocyte activation in the cerebellum through immunohistochemistry with GFAP. In Figure 3a, an increase of GFAP staining in cerebellum of hyperammonemic rats is shown, whereas bicuculline treatment reversed this increase. Quantification of the GFAP-stained area (Figure 3b) showed that it was significantly increased in hyperammonemic rats (26 ± 0.6 µm^2^, *p* < 0.001) compared to controls (22 ± 0.4 µm^2^) but not in control or hyperammonemic rats treated with bicuculline (23 ± 0.6 and 23 ± 0.6 µm^2^, respectively, *p* < 0.01 compared with hyperammonemia without bicuculline) (Welch ANOVA test: F (3.0, 336.6) = 11.17, *p* < 0.0001), indicating that bicuculline reversed astrocyte activation in the cerebellum of hyperammonemic rats. 

In order to understand whether bicuculline can restore the increase of pro-inflammatory cytokines in the cerebellum of hyperammonemic rats, we analyzed the content of IL-1β and of TNFα. 

**Figure 4:**
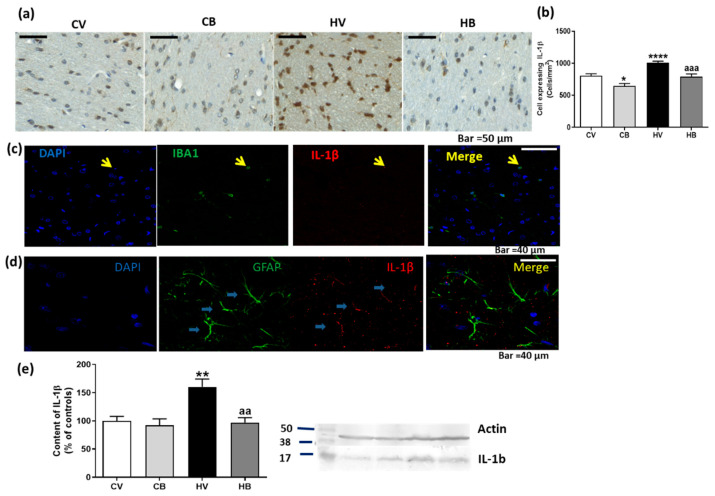
Effect of bicuculline on the content of IL1β in white matter of the cerebellum. Immunohistochemistry was performed using antibody against IL-1β. Representative images of IL-1β staining in white matter of the cerebellum are shown (**a**). The number of cells expressing IL-1β was quantified. Data are the mean ± SEM of 3–4 rats per group (**b**). Double immunofluorescence of IL-1β and Iba1 is shown in (**c**) and of IL-1β and GFAP in (**d**), confirming the expression of IL-1β in both microglia and astrocytes. The content of IL-1β was also analyzed by Western blot in the total cerebellum. Values are mean ± SEM of 12–14 samples per group (**e**). Values significantly different from control rats are indicated by asterisks: * *p* < 0.05, ** *p* < 0.01, **** *p* < 0.0001. Values significantly different from hyperammonemic rats are indicated by “aa”, *p* < 0.01, “aaa”, *p* < 0.001. CV, control vehicle; CB, control treated with bicuculline; HV, hyperammonemic rats with vehicle; HB, hyperammonemic rats treated with bicuculline.

IL-1β levels were assessed by immunohistochemistry in the white matter of the cerebellum (Figure 4a). Quantification of cells expressing IL-1β showed that the number of cells expressing IL-1β was significantly increased in hyperammonemic rats (1005 ± 29 cells/mm^2^, *p* < 0.0001) compared with controls (807 ± 31 cells/mm^2^). Bicuculline treatment significantly reversed IL-1β expression in hyperammonemic rats (794 ± 41 cells/mm^2^, *p* < 0.001, compared with hyperammonemia without bicuculline), confirming the results obtained by Western blot (Figure 4b). The parameters for the Brown–Forsythe ANOVA test were F (3.0, 178.7) = 17.54, *p* < 0.0001. To confirm expression of IL-1β in microglia or astrocytes, we performed a double immunofluorescence of IL-1β with GFAP or Iba1 in hyperammonemic rats. IL-1β was expressed in both astrocytes and in microglial cells (Figure 4c,d). The total content of IL-1β assessed by Western blot was significantly increased in the cerebellum of hyperammonemic rats (160 ± 14% of controls, *p* < 0.01). Bicuculline treatment did not alter the IL-1β content in control rats but significantly reversed the increase in hyperammonemic rats (97 ± 9% of controls, *p* < 0.01 compared with hyperammonemia without bicuculline) (F (3, 46) = 8.695), *p* < 0.0001) (Figure 4e).

**Figure 5:**
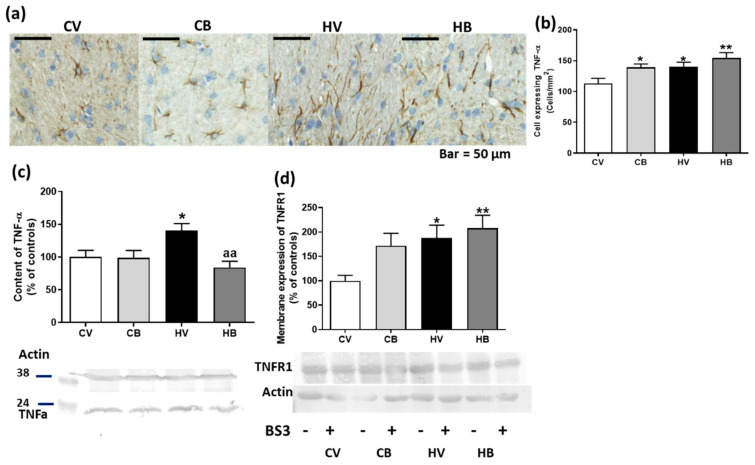
Effect of bicuculline on the content of TNFα and membrane expression of TNFR1 in the cerebellum. Immunohistochemistry was performed using the antibody against TNFα. Representative images of TNFα staining in the white matter of the cerebellum are shown in (**a**). The number of cells expressing TNFα was quantified. Data are the mean ± SEM of three rats per group (**b**). The content of TNFα was also analyzed by Western blot in the total cerebellum (**c**), along with the membrane expression of TNFR1 (**d**). Values are the mean ± SEM of 10–12 samples per group. Values significantly different from control rats are indicated by asterisks: * *p* < 0.05, ** *p* < 0.01. Values significantly different from hyperammonemic rats are indicated by “aa”, *p* < 0.01. CV, control vehicle; CB, control treated with bicuculline; HV, hyperammonemic rats with vehicle; HB, hyperammonemic rats treated with bicuculline.

The expression of TNFα was also assessed by Western blot and immunohistochemistry in white matter of cerebellum. The number of cells expressing TNFα increased in hyperammonemic rats treated with bicuculline or not (141 ± 7 cells/mm^2^ in HA, *p* < 0.05 and 154 ± 9 cells/mm^2^ in HA + BIC, *p* < 0.01) compared with control rats (113 ± 8 cells/mm^2^). In addition, bicuculline treatment also increased TNFα expression in control rats (139 ± 5 cells/mm^2^, *p* < 0.05) (F (3, 145) = 4.792, *p* < 0.01) (Figure 5a,b). However, transmembrane TNFα (26 kd isoform) content, analyzed by Western blotting, was significantly increased in hyperammonemic rats (141 ± 10% of controls, *p* < 0.05), but bicuculline treatment decreased its content to control values (84 ± 10% of controls, *p* < 0.01, compared with hyperammonemia without bicuculline). The content of transmembrane TNFα (26 KD) was not altered in control rats treated with bicuculline (99 ± 11% of controls) (F (3, 37) = 5.378, *p* < 0.01) (Figure 5c). Membrane expression of TNFR1 increased in the cerebellum of hyperammonemic rats, but bicuculline did not restore it (Figure 5d) (188 ± 26% of controls, *p* < 0.05 for hyperammonemic rats and 208 ± 27% of controls, *p* < 0.01 for hyperammonemic rats treated with bicuculline). In addition, bicuculline also increased the membrane expression of TNFR1 in control rats (172 ± 26% of controls) (F (3, 43) = 4.956, *p* < 0.01) (Figure 5d).

**Figure 6:**
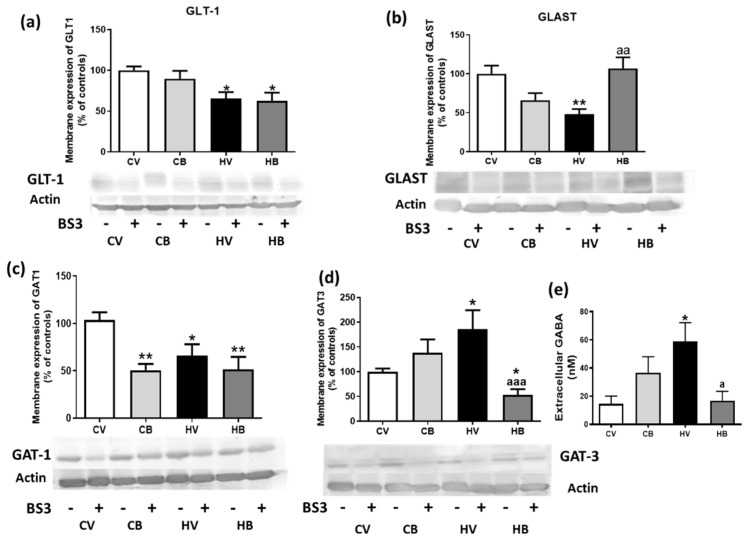
Effect of bicuculline on membrane expression of glutamate and GABA transporters and on extracellular GABA content. Membrane expression of glutamate transporters GLT-1 (**a**) and GLAST (**b**) and of GABA transporters GAT-1 (**c**) and GAT-3 (**d**) in the cerebellum was analyzed using the BS3 crosslinker procedure, as described in the Methods section. Representative images are shown. The extracellular concentration of GABA was measured by HPLC-MS in samples obtained by in vivo microdialysis in the cerebellum (**e**). Values are expressed as percentage of control rats and are the mean ± SEM of 8–12 rats per group. Values significantly different from control rats are indicated by asterisks: *p < 0.05, ** *p* < 0.01 and from hyperammonemic rats by “a”: a, *p* < 0.05; aa, *p* < 0.01; aaa, *p* < 0.001.

Previously, we reported an association between neuroinflammation and the membrane expression of glutamate and GABA transporters in the cerebella of hyperammonemic rats. In hyperammonemic rats, membrane expression of glutamate transporters was significantly decreased (66 ± 8%, *p* < 0.05 of controls for GLT-1 and 48 ± 7% of controls, *p* < 0.01 for GLAST) (Figure 6a,b) according to previous results. Bicuculline restored membrane expression of GLAST in hyperammonemic rats (107 ± 14% of controls, *p* < 0.01 with respect to hyperammonemia without bicuculline) but not those of GLT-1 (63 ± 10% of controls, *p* < 0.01) (Figure 6a,b). Statistical analysis with one-way ANOVA showed the following parameters: GLT-1, F (3, 47) = 5.468, *p* < 0.01, and GLAST, F (3, 24) = 7.854, *p* < 0.001. In addition, GABA transporters were also altered. Membrane expression of GABA transporter GAT-1 was significantly decreased (66 ± 12% of controls, *p* < 0.05) in hyperammonemic rats (Figure 6c). Moreover, GAT-3 was significantly increased in hyperammonemic rats (161 ± 34% of controls, *p* < 0.05) (Figure 6d); this was related to a reversal of its function, indicating that there was a release of GABA through this transporter instead of an uptake. Bicuculline effectively decreased the membrane expression of GAT-3 (53 ± 12% of controls, *p* < 0.001) (Figure 6d) but did not reverse the decrease of membrane expression of GAT-1 in hyperammonemic rats (51 ± 13% of controls, *p* < 0.01) (F (3, 38) = 7.598, *p* < 0.001). In addition, bicuculline also decreased membrane expression of GAT-1 in control rats (51 ± 6% of controls, *p* < 0.01) (F (3, 38) = 6.969, *p* < 0.001) (Figure 6c). Both alterations led to increased extracellular GABA (59 ± 13 nM, *p* < 0.05, compared with 14.4 ± 6 nM in control rats) (Figure 6e). The extracellular concentration of GABA was decreased by bicuculline in hyperammonemic rats (17 ± 7% nM, *p* < 0.05 compared with hyperammonemia without bicuculline) (F (3, 21) = 4.329, *p* < 0.05) (Figure 6e), surely due to a reversal of the increase of GAT-3 membrane expression, as GAT-1 remained decreased in hyperammonemic rats treated with bicuculline. An increase in extracellular GABA, but not a significant one, was observed in control rats treated with bicuculline.

**Figure 7:**
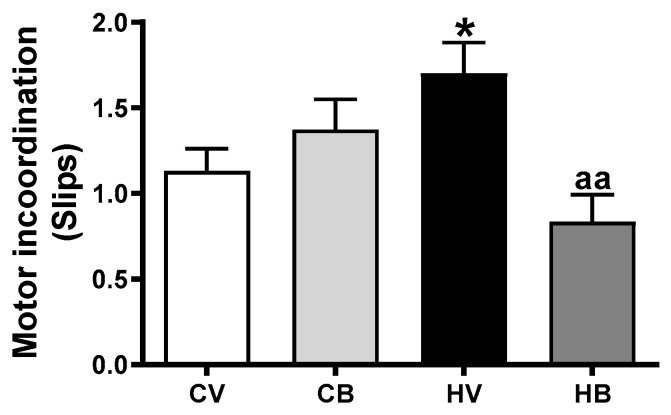
Effects of bicuculline on motor coordination with the beam walking test. Motor coordination was assessed with the beam walking test as described in the Methods section. The graph shows *Mean±SEM of 12* rats per group. Values significantly different from control rats are indicated by asterisks and from hyperammonemic rats by “a”, * *p* < 0.05; aa, *p* < 0.01.

As increased extracellular GABA and activation of GABA_A_ receptors induce motor incoordination, we confirmed that blocking GABA_A_ receptors with bicuculline administration improved motor coordination in hyperammonemic rats. As shown in Figure 7, hyperammonemic rats showed higher number of slips (1.7 ± 0.2 slips, *p* < 0.01) than control rats (1.1 ± 0.1 slips). The number of slips was significantly decreased in hyperammonemic rats treated with bicuculline (0.8 ± 0.2, *p* < 0.01, compared with hyperammonemic rats without bicuculline) (F (3, 76) = 4.116, *p* < 0.01). As indicated by the increase in extracellular GABA in the cerebellum, some motor incoordination (but not a significant effect) was observed in control rats treated with bicuculline (Figure 7).

**Figure 8:**
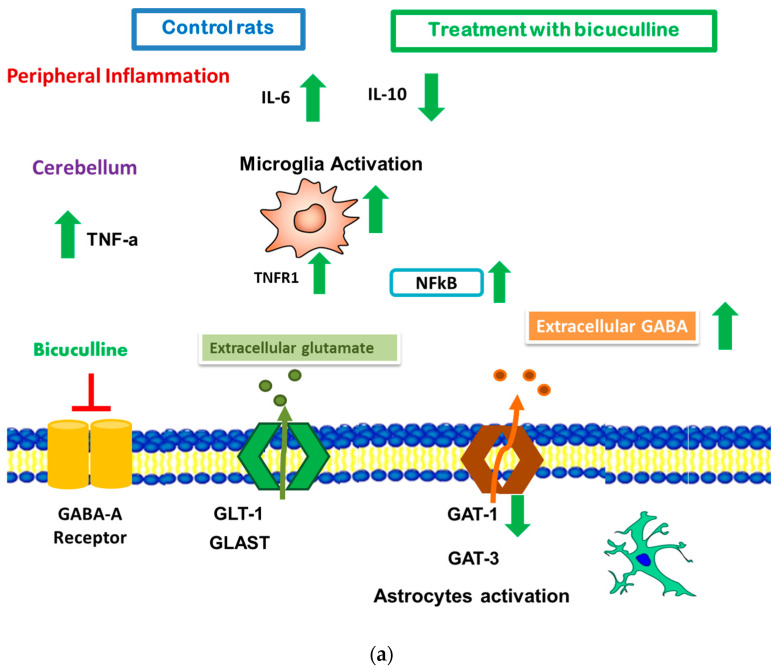

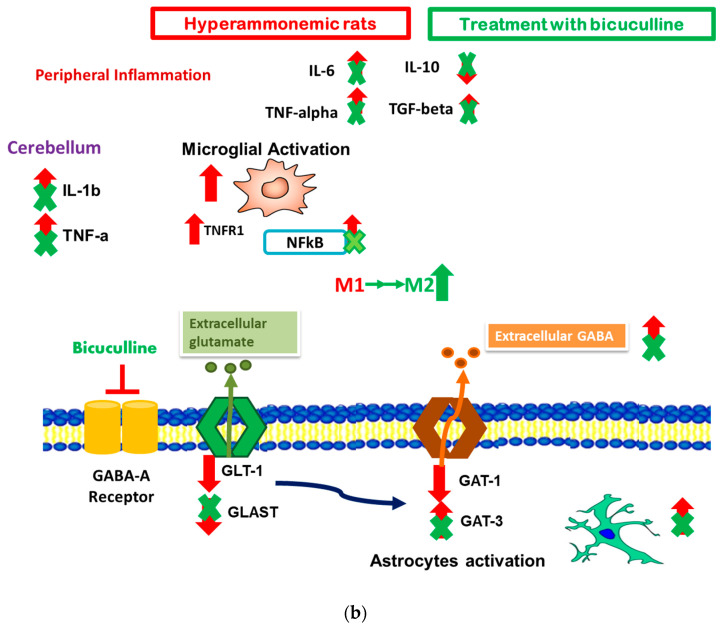
Scheme showing the effects of blocking GABA_A_ receptors with bicuculline on peripheral and cerebellar neuroinflammation and neurotransmission. (**a**) Control rats. Chronic treatment with bicuculline induced a peripheral pro-inflammatory environment shown by an increase in IL-6 and a decrease in IL-10. In the cerebellum, bicuculline also induced neuroinflammation, increasing TNFα levels and inducing NFkB in activated microglia. In addition, GABA neurotransmission was also impaired by decreasing the GAT-1 membrane expression, which led to a weak increase in extracellular GABA and motor incoordination. (**b**) Hyperammonemic rats. In hyperammonemic rats, bicuculline prevented the increase of the pro-inflammatory cytokines IL-6 and TNFα, increased IL-10, and decreased TGF-β levels in plasma, improving peripheral inflammation. In the cerebellum, bicuculline induced a change in the phenotype of microglia to a more anti-inflammatory state, M2, decreasing NFkB nuclear localization, but did not affect the increased membrane expression of TNFR1. Bicuculline prevented astrocyte activation and decreased the content of the pro-inflammatory cytokines TNFα and IL-1β. Bicuculline restored the membrane expression of GLAST and decreased the membrane expression of GAT-3 without any effect on GLT1 and GAT1 transporters. Even so, extracellular levels of GABA were also returned to control levels by bicuculline. This suggests that bicuculline should mainly affect peripheral inflammation and astrocyte activation, possibly through a direct effect on GABAA receptors in peripheral immune cells and in astrocytes.

A schematic summary of the main findings in control (a) and hyperammonemic rats (b) is shown in Figure 8.

## 3. Discussion

We analyzed the effects of blocking GABA_A_ receptors with the chronic systemic administration of bicuculline on inflammatory markers in blood and in the cerebellum, both in control and in hyperammonemic rats, the last showing peripheral inflammation and neuroinflammation. 

Hyperammonemic rats showed increased extracellular GABA in the cerebellum, which increased the activation of GABA receptors, both GABAA and GABAB. In this work, we analyzed only the effect of blocking GABAA, but effects of GABAB receptors activation on inflammation are also reported in the literature [24,25,26]. Therefore, future research can address the role of GABAB receptors on inflammation and neuroinflammation in hyperammonemic rats.

**Figure 9:**
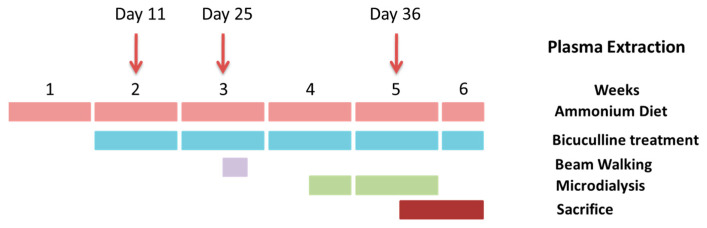
Scheme showing the experimental design.

It is widely reported that GABA_A_ activation has anti-inflammatory effects in some pathological situations [16,27,28,29,30]. We found that the chronic inhibition of the GABA_A_ receptor in control rats induced slight peripheral inflammation, increasing IL-6 and decreasing IL-10. In addition, bicuculline also induced microglial activation, NFkB nuclear localization, and an increase in the membrane expression of TNFR1 and in TNFα in the white matter of the cerebellum from control rats (Figure 9). This suggests that, in physiological conditions, not only in inflammatory pathologies but also the GABA system modulate the immune system, as was shown in [31], where an increase in TNFα in naïve macrophages exposed to picrotoxin was found.

However, bicuculline improved inflammatory parameters in hyperammonemic rats, decreasing IL-6 and TNFα and increasing IL-10 levels in the plasma. A similar effect of intracisternal administration of bicuculline was found by Sallam et al. [18], which showed a decrease in serum levels of IL-6 and TNFα in rats injected intraventricularly with LPS after bicuculline injection. These data suggest that the effect of bicuculline on peripheral inflammation could be due to its effect on brain receptors.

The inflammatory effects of GABA and anti-inflammatory effects of GABA_A_ receptor inhibition were also observed previously by other authors in hepatic injury [32], endotoxemia [18], or autoimmune encephalomyelitis [17]. Different mechanisms for the opposed function of GABA on different inflammatory aspects were proposed. Carmans et al. [17] suggested that endogenous GABA improves inflammation, whereas exogenous GABA enhances it, in a model of autoimmune encephalomyelitis. Lee et al. [33] reported an enhancement of inflammatory hypersensitivity by GABA through peripheral GABA_A_ receptor activation. It was suggested that this dual action of GABA_A_ receptors was due to different subunit composition in stress-induced gut inflammation [34]. 

Carmans et al. [17] showed that systemic GABA induced proliferation of T cells and increased IFNγ, whereas it decreased IL-4 levels in splenocytes, inducing a Th1 response. We can suggest that bicuculline can inhibit the GABA induction of Th1, decreasing the ratio Th1/Th2. Th2 production of IL-10 and TGFβ may induce M2 phenotype in macrophages, contributing to the decrease in TNFα production. 

On the other hand, the activation of regulatory T-lymphocytes (T-regs) also increased both IL-10 and TGF-β [35,36]. Our results showed a clear increase in TGF-β when IL-10 was decreased in hyperammonemic rats, suggesting that the increase in TGF-β can be induced from a different source than the activation of T-regs. A possible explanation for this situation is that, since liver damage is an important source of TGF-β [37], and hyperammonemia is thought to impair liver function [38,39], this can contribute to the increase in TGF-β in plasma. In hyperammonemic rats, both cytokines increased after four weeks of chronic treatment with bicuculline, suggesting that bicuculline could activate T-regs in these rats, thereby inducing an increase in both IL-10 and TGF-β. Augmented levels of these anti-inflammatory cytokines may lead to the decrease in TNFα. The increase in IL-10 and the decrease in TNFα serum levels by bicuculline were also reported by Hsu et al. [32]. These authors showed a beneficial effect of bicuculline treatment on hepatic injury in a model with sepsis and suggested that this effect should be mediated by modulation of cytokine production, which agrees with the results presented here.

In the cerebellum, the chronic administration of bicuculline decreased astrocyte activation, induced polarization of microglia to an anti-inflammatory phenotype M2, and decreased IL-1β content (Figure 8b). The effect of bicuculline on peripheral cytokines could contribute to the decrease in cerebellar neuroinflammation, as neuroinflammation is due to peripheral inflammation. Blocking peripheral TNFα signaling with infliximab prevented neuroinflammation, preventing microglia and astrocyte activation and the increase in IL-1β [5,40]. However, we cannot ignore that a direct effect of bicuculline on cerebellar receptors can also lead to inhibition of astrocyte activation as well as to the decrease in IL-1β and the change in microglia phenotype in hyperammonemic rats. A direct effect of GABA on astrocyte morphology increasing branching and GFAP expression was reported [21]. Therefore, we can suppose that the observed effect of bicuculline on astrocyte activation can be mediated, at least partially, by direct decrease of GABA signaling. 

Bicuculline does not restore TNFα content in the white matter, but its levels were decreased in the total cerebella of hyperammonemic rats treated with bicuculline. This discrepancy can be explained by the decrease in peripheral TNFα or in the content of TNFα in other cerebellar layers but not of TNFα expression in white matter, as the whole cerebellum, including endogenous capillaries, was analyzed by Western blot. 

A complex role of GABA_A_ in macrophage polarization was also reported [31]. These authors showed that inflammation induced by LPS also modulated the GABA system in macrophages, showing a bidirectional modulation between GABA signaling and immune cells function. Our results also suggest an effect of GABA_A_ on microglia phenotype and a possible effect of microglia on GABA system, as bicuculline activated microglia in control rats and increased GABA extracellular levels by decreased membrane expression of GAT-1, whereas, in hyperammonemic rats showing neuroinflammation, bicuculline induced M2 phenotype in microglia and decreased GABA extracellular levels, decreasing membrane expression of GAT-3 and astrocyte activation. 

Bicuculline restored the membrane expression of GLAST and GAT3 but not of GLT-1 and GAT1. This suggests that the membrane expression of GLAST and GAT3 should be modulated by astrocyte activation and IL1β increase, whereas the membrane expression of GAT1 and GLT1 should be only modulated by TNFα or directly modulated by GABA_A_ inhibition by bicuculline with a small neuroinflammation effect. This observation is in accordance with the effect of sulforaphane in the cerebellum of hyperammonemic rats, since sulforaphane also inhibited the increase in membrane expression of GAT3, the activation of astrocytes, and induced a change in the microglia phenotype from M1 to M2, leading to an improvement in motor coordination. Sulforaphane also improved learning ability in the Y maze, as was previously reported for bicuculline chronic treatment [7]. This suggests that future research should analyze some possible anti-oxidant effects of bicuculline as well as the effect of bicuculline on the Nrf2 signaling pathway, as sulforaphane exerts its effect through this pathway. Kochiyama et al. [41] showed the modulation of Nrf2 by GABA_A_ receptors. It was shown that the activation of TNFR1 induced NFkB nuclear translocation in hyperammonemic rats [6]. Bicuculline did not prevent the membrane expression of TNFR1 but it prevented NFkB nuclear localization, indicating that its effect could be mediated by the inhibition of a downstream step. Ferrari et al. [42] reported that Nrf2 activation prevented NFkB activation by TNFα [42]. Therefore, this may also be the possible mechanism of inhibition of NFkB by bicuculline.

Our results also show that decreasing the peripheral levels of TNFα in chronic hyperammonemia did not prevent all the effects on cerebellar neuroinflammation and neurotransmission but should be necessary to prevent the initial increase of TNFα in plasma, as, in this work, bicuculline treatment started after one week of hyperammonemia, when cerebellar neuroinflammation was already observed [5], whereas, in our previous works, Infliximab treatment started before hyperammonemic diet [5,40].

Our results suggest that bicuculline-induced peripheral inflammation, microglial activation in the cerebellum, and a decrease in the membrane expression of GAT-1, leading to a small increase in extracellular GABA, were not sufficient to lead to significant impairment of motor coordination in control rats (Figure 8a). However, in hyperammonemic rats, which also showed astrocyte activation and decrease in GLAST and GLT1 and increase in GAT-3 membrane expression, extracellular GABA levels were higher, leading to motor coordination impairment (Figure 8b).

We want to point out that bicuculline treatment, by blocking GABAA receptors, can directly improve motor incoordination in hyperammonemic rats, but our results show that, in addition, bicuculline decreased extracellular GABA levels via modulation of the membrane expression of GABA transporters. 

## 4. Materials and Methods

Study design: chronic hyperammonemia in rats and treatment with bicuculline. Male Wistar rats (120–140 g, Charles River Laboratories, Barcelona, Spain) were made hyperammonemic by feeding them an ammonium-containing diet (ammonium acetate 30%), as in [43]. Animals were distributed into four groups: control with vehicle (CV); control treated with bicuculline (CB); hyperammonemic rats (HA); hyperammonemic rats treated with bicuculline (HB). Bicuculline ((+)-Bicuculline, Sigma-Aldrich, Saint Louis, MI, USA) was injected intraperitoneally, 0.3 mg/kg, once per day. The dose was chosen based on a previous study of the use of bicuculline in hyperammonemic rats [7]. Bicuculline was dissolved in physiological serum (NaCl 0.9%) with 0.3% DMSO, and this solution was used as vehicle.

The experiment was repeated four times, and nine animals per group were used in each experiment (a total of 36 rats per group). The experimental design is summarized in Figure 9. The experiments were approved by the Comité de Ética y Experimentación Animal, Prince Felipe Research Center-Consellería de Agricultura, Generalitat Valenciana and were carried out in accordance with the Directive of the European Commission (2010/63/EU) for care and management of experimental animals and complied with the ARRIVE guidelines for animal research. 

Measurement of ammonia and cytokines in plasma. Blood was collected from the saphenous vein on days 11, 25, and 36 after starting the hyperammonemic diet. For plasma preparation, blood was collected in EDTA-containing vials, centrifuged at 6000× *g* for 5 min, and stored at −80 °C. Twenty microliters of blood were immediately used to measure ammonia with the Ammonia Test Kit II for the PocketChemBA system (Arkay, Inc., Kyoto, Japan). 

TNFα in plasma was measured using an ELISA Kit from eBioscience (San Diego, CA, USA). The content of all other cytokines in plasma was analyzed by Western blot using primary antibodies against IL-10, TGF-β (1:1000) from Abcam (Cambridge, UK), and IL-6 (1:500) from MyBioSource (San Diego, CA, USA). Secondary antibodies were anti-rabbit or anti-mouse (1:4000) IgG, conjugated with alkaline phosphatase (Sigma). The images were captured using a Hewlett Packard (San José, CA, USA) ScanJet 5300C, and the band intensities were quantified using the AlphaImager 2200 software (AInnotech Corporation, San Francisco, CA, USA).

Brain immunohistochemistry and immunofluorescence. At week 5 of hyperammonemia, the rats were anaesthetized with sodium pentobarbital and transcardially perfused with 0.9% saline, followed by 4% paraformaldehyde in a 0.1 M phosphate buffer (pH 7.4). Brains were removed and post-fixed in the same fixative solution for 24 h at 4 °C. Paraffin-embedded sections (5 µm) were cut and mounted on coated glass slides. The tissue sections were then processed with the Envision Flex + kit (DAKO, Santa Clara, CA, USA), blocking endogenous peroxidase activity for 5 min, and then incubated with antibodies. Primary antibodies were against Iba-1 (Wako 019-19741; 1:300 for 30 min), Glial Fibrillary Acidic Protein (GFAP) (Dako IR524; ready for use for 20 min), IL-1β (Abcam AB9722; 1:100 dilution for 30 min), and TNFα (Abcam; 1:2000 for 45 min). The reaction was visualized by incubation with Envision Flex plus horseradish peroxidase for 20 min and, finally, diaminobenzidine for 10 min. Sections were counterstained with Mayer’s hematoxylin for 5 min.

A double immunofluorescence was performed to confirm IL1b co-localization with microglia (using Iba1, 1:300, Abcam) and astrocytes (using GFAP, Sigma, 1:400).

Analysis of microglial activation. Analysis of Iba-1-stained microglia was performed in the cerebellum white matter using ImageJ 1.48v software. Microglial activation was assessed by measuring the cell perimeter in eight randomly selected areas (0.45 mm^2^) per section, according to [44]. The area of interest was selected. Using Auto Local Threshold and Analyze particle functions in ImageJ, the intensity thresholds and the size filter were applied. To measure the perimeter of microglia, the Bernsen method was used, and a 2000–20,000 size filter was applied. For each rat, at least 30–40 cells were quantified, and the results were converted from pixels to micrometers. 

Analysis of astrocyte activation. The astrocytic area (µm^2^) covered by GFAP was measured using the ImageJ software. Using the Auto Local Threshold and the Analyze Particles functions, the intensity thresholds and the size filter were applied. To measure the total cell size, the Bernsen method was used, and a 1500–7000 size filter was applied. For each rat, at least 20 cells from three different sections were counted. 

Analysis of NF-κB p50 nuclear localization in microglia. An analysis of the expression of the p50 subunit of NF-κB in microglia was performed by double immunofluorescence. Sections from six different animals per group were selected, washed in 0.1 M phosphate buffer, and blocked with normal serum from the same species as the secondary antibody before being incubated overnight with a primary antibody (NF-κB p50, 1:200; Iba1, 1:300) from Abcam, diluted in blocking buffer and secondary fluorescent antibodies (1:400) from Invitrogen (Carlsbad, CA, USA). The nuclei were counterstained with DAPI (Sigma-Aldrich) and the sections cover-slipped. The images were observed under a confocal microscope (TCS-SP2-AOBS, Leica (Wetzlar, Germany) and photographically recorded.

The cells positive for NFkB p50 were counted and are expressed as cells/mm^2^. The p50 subunit may be located in the nuclei or in the cytosol. The nuclear and cytoplasmic intensity of the p50 subunit was analyzed using ImageJ (1.48 v). Nuclei were outlined using the ROI manager function on the DAPI blue channel, and the selection was applied on the green channel (p50) to measure fluorescence. The mean intensity (MI) for each nucleus was measured. For cytoplasmic analysis of the p50 subunit of NF-κB, green channels were used, the cytosol of each cell was manually outlined using a freehand selection of ImageJ, and MI was recorded. Results are expressed as the percentage of cells expressing the p50 subunit of NF-κB in the nucleus. 

Analysis of protein content in cerebellum by Western blot. Homogenates of cerebellum were subjected to immunoblotting. Primary antibodies were against IL-1β (1:500 dilution (AF-510-NA) from R&D SYSTEMS, Minneapolis, MN, USA), TNFα (1: 500, R&D SYSTEMS), and YM-1 (1:500, Abcam). Secondary antibodies were anti-rabbit, anti-goat, or anti-mouse IgG, 1:4000 dilution (cat. #A8025, A7650, and A3562, respectively), conjugated with alkaline phosphatase from Sigma (St. Louis, MO, USA). The images were captured using ScanJet 5300C (Hewlett-Packard, Amsterdam, the Netherlands) and the band intensities quantified using AlphaImager 2200, version 3.1.2 (Alpha Innotech, Watertown, MA, USA).

Analysis of membrane surface expression of GABA and glutamate transporters and of TNFR1. Cerebella were dissected, and transversal slices (400 µm) were obtained using a chopper. Slices were added to tubes containing ice-cold standard buffer with or without 2 mM BS3 (crosslinker) (Pierce, Rockford, IL, USA) and incubated for 30 min at 4 °C. Cross-linking was terminated by adding 100 mM glycine (10 min, 4 °C). The slices were homogenized by sonicating for 20 s. Samples treated with BS3 or not were analyzed by Western blot. The antibodies used were: anti-GLT1 (1:1000, Invitrogen), anti-GLAST (1:4000, Novus Biologicals (Centennial, CO, USA)), anti-GAT1 (1:500, Abcam), anti-GAT3 (1:500, Millipore (Burlington, MA, USA)), and anti-TNFR1 (1:1000, Abcam). The membrane surface expression of each receptor was calculated as the difference between the intensity of the bands without BS3 (total protein) and with BS3 (cytosolic protein), as described by [45]. 

In vivo cerebellar microdialysis and determination of extracellular GABA. Rats were anesthetized using isoflurane, and a microdialysis guide was implanted in the cerebellum (AP—10.2, ML—1.6, and DV—1.2), as in [46]. After 48 h, a microdialysis probe was implanted in the freely moving rat. Probes were perfused (3 μL/min) with artificial cerebrospinal fluid (in mM): NaCl, 145; KCl, 3.0; CaCl_2_, 2.26; buffered at pH 7.4 with 2 mM phosphate. After a 2 h stabilization period, samples were collected every 30 min. EDTA was added to the samples at a final concentration of 4 mM and stored at −80 °C until analysis of GABA levels. To assess the basal level of extracellular GABA in the cerebellum, five consecutive microdialysis samples were collected from each rat. The extracellular concentration of GABA was measured by HPLC-MS, as in [47]. 

Motor coordination in the beam-walking test. The beam-walking test consists of a wood strip 20 mm in diameter and 1 m long, located 1 m above the ground. Rats have to cross the beam, and two observers count the number of slips committed by the rats, as described in [8]. The average number of foot faults (slips) observed by two experimenters is recorded as a measure of incoordination.

Statistical analysis. Data are expressed as mean ± SEM. All statistical analyses were performed using GraphPad Prism 7.0 (GraphPad Prism Software, Inc., San Diego, CA, USA). All data were tested for normality with D’Agostino and Pearson’s tests. When the data did not pass a normality test, Kruskal–Wallis’s test with Dunn’s test for multiple comparisons were performed. If the data showed normal distribution, we used one-way ANOVA with Tukey’s test for multiple comparisons or Welch’s test with Dunnett’s test when the deviations were not equal. To test the progression of cytokine levels in the plasma, two-way ANOVA with repeated measures followed by Bonferroni’s test for multiple comparisons was performed. A confidence level of 95% was considered significant.

## 5. Conclusions

In summary, we showed here that the inhibition of GABA_A_ receptors with the systemic administration of bicuculline altered peripheral inflammatory markers in control rats, inducing a pro-inflammatory environment, whereas, in hyperammonemic rats with a previous pro-inflammatory environment, bicuculline prevented it, leading to a more anti-inflammatory environment. In the same way, in the cerebellum, bicuculline increased microglial activation in controls, while, in hyperammonemic rats, bicuculline induced a microglial phenotype change to M2 (anti-inflammatory) and inhibited astrocyte activation. This modulation of the immune system is associated with altered membrane expression of the GABA transporters and GABA levels in both control and hyperammonemic rats, supporting the close interplay between GABAergic neurotransmission and immune modulation. Thus, our results bring new data on this interplay. 

On the other hand, this work also supports the beneficial role of bicuculline in cerebellar neuroinflammation associated with restored GABA neurotransmission and improvement of motor incoordination in hyperammonemic rats.

## Data Availability

Data are contained within the article. The data presented in this study are available in this article: The dual role of the GABA_A_ receptor in peripheral inflammation and neuroinflammation: a study in hyperammonemic rats.
